# Nordic consensus statement on the systematic assessment and management of possible severe asthma in adults

**DOI:** 10.1080/20018525.2018.1440868

**Published:** 2018-03-06

**Authors:** Celeste Porsbjerg, Charlotte Ulrik, Tina Skjold, Vibeke Backer, Birger Laerum, Sverre Lehman, Crister Janson, Thomas Sandstrøm, Leif Bjermer, Barbro Dahlen, Bo Lundbäck, Dora Ludviksdottir, Unnur Björnsdóttir, Alan Altraja, Lauri Lehtimäki, Paula Kauppi, Jussi Karjalainen, Hannu Kankaanranta

**Affiliations:** ^a^ Institute of Clinical Medicine, University of Copenhagen, Copenhagen, Denmark; ^b^ Respiratory Research unit, Department of Respiratory Medicine, Bispebjerg Hospital, Copenhagen, Denmark; ^c^ Department of Respiratory Medicine, Hvidovre Hospital, Hvidovre, Denmark; ^d^ Dept of Respiratory Medicine, Aarhus University Hospital, Aarhus C, Denmark; ^e^ LHL-klinikkene Bergen, Nesttun, Norway; ^f^ Department of Clinical Science, University of Bergen, Bergen, Norway; ^g^ Department of Thoracic Medicine, Haukeland University Hospital, Bergen, Norway; ^h^ Department of Medical Sciences: Respiratory, Allergy & Sleep Research, Uppsala University, Uppsala, Sweden; ^i^ Department of Public Health and Clinical Medicine, Division of Medicine, Umeå University, Umeå, Sweden; ^j^ Department of Respiratory Medicine & Allergology, Skåne University Hospital, Lund, Sweden; ^k^ Division of Respiratory Medicine and Allergy, Karolinska University Hospital, Stockholm, Sweden; ^l^ Institute of Medicine/Krefting Research Centre University of Gothenburg, Gothenburg, Sweden; ^m^ Dept. of Allergy, Respiratory Medicine and Sleep Landspitali University Hospital Reykjavik Iceland, University of Iceland, Reykjavik, Iceland; ^n^ Faculty of Medicine, University of Iceland, Reykjavik, Iceland; ^o^ Department of Pulmonary Medicine, University of Tartu and Department of Pulmponary Medicine, Tartu University Hospital, Tartu, Estonia; ^p^ Faculty of Medicine and Life Sciences, University of Tampere, Tampere, Finland; ^q^ Allergy Centre, Tampere University Hospital, Tampere, Finland; ^r^ Department of Allergy, Respiratory Diseases and Allergology, University of Helsinki and Helsinki University Hospital, Helsinki, Finland; ^s^ Department of Respiratory Medicine, Seinäjoki Central Hospital, Seinäjoki, Finland

**Keywords:** Asthma, severe, prevalence, diagnosis, co-morbidities, management, guideline

## Abstract

Although a minority of asthma patients suffer from severe asthma, they represent a major clinical challenge in terms of poor symptom control despite high-dose treatment, risk of exacerbations, and side effects. Novel biological treatments may benefit patients with severe asthma, but are expensive, and are only effective in appropriately targeted patients. In some patients, symptoms are driven by other factors than asthma, and all patients with suspected severe asthma (‘difficult asthma’) should undergo systematic assessment, in order to differentiate between true severe asthma, and ‘difficult-to-treat’ patients, in whom poor control is related to factors such as poor adherence or co-morbidities. The Nordic Consensus Statement on severe asthma was developed by the Nordic Severe Asthma Network, consisting of members from Norway, Sweden, Finland, Denmark, Iceland and Estonia, including representatives from the respective national respiratory scientific societies with the aim to provide an overview and recommendations regarding the diagnosis, systematic assessment and management of severe asthma. Furthermore, the Consensus Statement proposes recommendations for the organization of severe asthma management in primary, secondary, and tertiary care.

## Introduction

Although the majority of asthma patients have mild to moderate disease, a proportion of asthma patients have difficulty in achieving control on standard treatment or require very high doses of treatment to maintain asthma control, and risk of side effects [1,2]: In the patients with severe asthma, control is not obtained despite correction of comorbidities and correct use of high doses of asthma medications. Patients with severe asthma represent a major unmet need, as they experience frequent exacerbations, are hospitalized more often, and utilize the majority of health care expenses in asthma [3].

There are a number of potential causes of poor symptom control in asthma [4], and systematic assessment is important when differentiating between patients with severe asthma, and patients with other causes of poor asthma control, such as lack of adherence or co-morbidities, termed ‘difficult-to-treat’ asthma.

A number of novel treatments for severe asthma are under development, some of which have been approved for clinical use: treatment with anti-IgE and anti-IL5 monoclonal antibodies are effective in reducing the risk of asthma exacerbations [5,6]. However, as the biological treatments target very specific pathways in the immune system, they are only effective in specific phenotypes of severe asthma [7]. Accordingly, phenotyping of severe asthma patients has become increasingly important, in order to target novel treatments tailored to the appropriate patient.

Systematic assessment and phenotyping of patients with possible severe asthma requires a highly specialized setting, to ensure an appropriate and effective diagnostic work-up [8]. The Nordic Countries share similar healthcare systems, as well as a similar demography: Based on the reported prevalence rates of severe asthma [9,10], approximately 33,000 patients among the 22 million inhabitants of the Nordic countries can be estimated to suffer from severe asthma.

The Nordic Severe Asthma Network (NSAN) was established under NORA, the Nordic Respiratory Societies in 2016, and consists of severe asthma specialists from Iceland, Norway, Sweden, Denmark, Finland, and Estonia. The aim of the NSAN is to increase awareness of severe asthma in the Nordic Countries, as well as improving the standard of care by providing guidelines on the management of severe asthma. The present Nordic consensus statement on severe asthma aims to provide pragmatic, clinically useful guidance on how to approach the patients with possible severe asthma; how to perform systematic assessment, how to identify potential candidates for biological treatments, and how to organize a severe asthma clinic.

## The definition of severe asthma

The ERS/ATS guidelines on severe asthma published in 2014 define severe asthma as ‘*asthma which requires treatment with high dose inhaled corticosteroids (ICS) plus a second controller or systemic CS, which remains “uncontrolled” despite this therapy, or to prevent it from becoming “uncontrolled”’* [1]. Hence, this definition includes patients, who are well controlled on high-dose therapy, but lose symptom control when down-titrated [1]. The definitions of high dose ICS are summarized in Table 1. ‘Second controllers’ include long-acting beta-2 agonist, leukotriene antagonists, long-acting anti-cholinergics or methylxantines.

**Table 1.** Definitions of high dose inhaled steroids (ICS)*.

Name	Daily dose (μg)*
Budesonide	≥1600
Fluticasone dipropionate	≥1000
Mometasone furoate	≥800
Beclomethason dipropionate	≥2000 (DPI or CFC MDI)
Ciclesonide	≥320
Fluticasone furoate	≥184
Triamcinolone acetonide	≥2000

* According to the ERS/ATS guidelines on severe asthma [1].

**Table 2.** Co-morbidities in severe asthma: diagnosis and management.

Co-morbidity	Prevalence	Test	Management
Rhinosinusitis/Nasal polyps	50% [61]	SNOT-22 questionaire CT of sinuses Nasendoscopy (ENT assessment)	Nasal lavage Nasal steroid spray/drops Surgery
Allergic Rhinoconjuctivitis (*Positive skin prick test to aeroallergens)	70% [61]*	History + skin prick test/specific IgE	Nasal steroids Antihistamines Montelukast
COPD	20% [171]	History incl smoking DLCO/HRCT (emphysema)	Add LAMA Add Roflumilast Rehabilitation
Dysfunctional Breathing	19–52 [59,172]%	History/Nijmegen questionaire	Physioterapy – breathing retraining
VCD	32–50% [59,74]-	Laryngoscopy	Speech therapist
Anxiety/Depression	4–17% [9,17]	HADS questionnaire Psychiatric assessment Psychiatrist	Medical treatment Psychotherapy
OSAS	31% [80]	Screening with STOP-BANG Polysomnography/respiratory polygraphy	Weight loss CPAP
Obesity	37% [18]	BMI	Dietician
Gastro-esophageal Reflux	17–74% [18,22,61,173]	3 months of empiric PPI 24-hours pH monitoring	PPI Lifestyle interventions
Bronchiectasis	25–40% [91,92]	HRCT	Physiotherapy, inhalation of hyperosmolar agents, low-dose macrolides
ABPA	1–2% [96]	Total IgE, IgE and IgG to aspergillus fumigatus, B-eosinophils, HRCT	Prednisolone. Anti-fungal treatment.

OSAS: Obstructive Sleep Apnea Syndrome. VCD: Vocal Chord Dysfunction. ABPA: Allergic Bronchopulmonary Aspergillosis.

**Figure 1.**
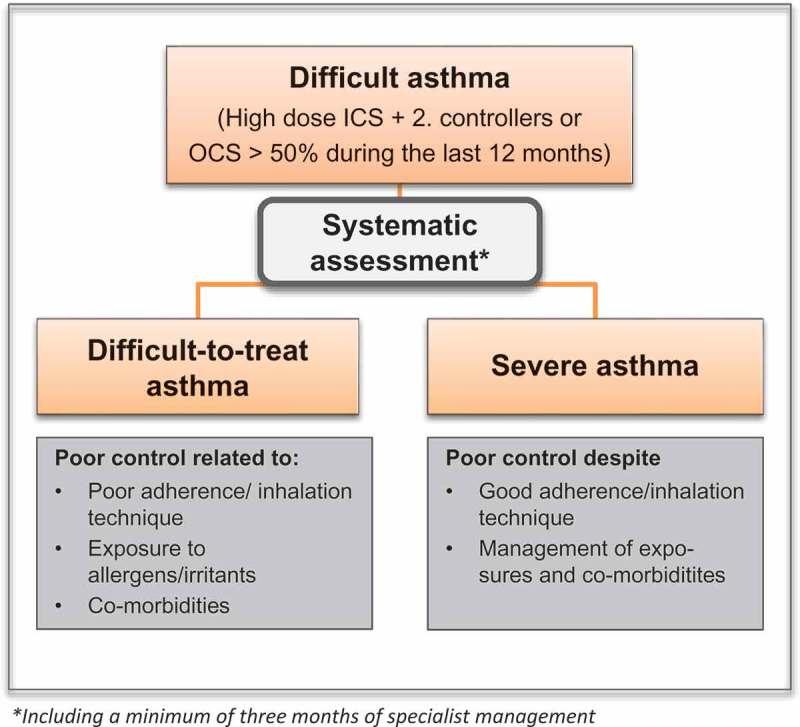
Severe asthma: definition and systematic assessement.

However, before a diagnosis of severe asthma can be made, patients need to undergo a systematic assessment: The ERS/ATS guidelines state that in patients with ‘difficult asthma’, (high dose ICS treatment + a second controller), *the diagnosis of asthma should be confirmed, and comorbidities addressed, before a diagnosis of severe asthma can be made* (Figure 1). Patients in whom poor asthma control is related to other factors, such as poor adherence or co-morbidities, are termed ‘difficult-to-treat asthma’^1^ (Figure 1).

## Systematic assessment of severe asthma

**Figure 2.**
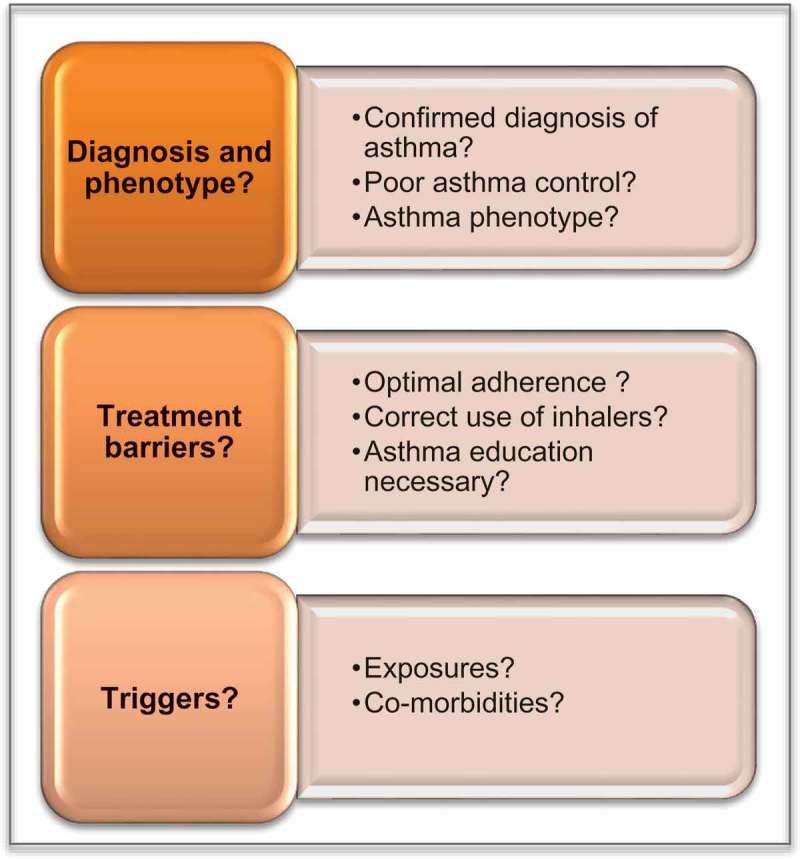
Systematic assessment of possible severe asthma.

[Systematic assessment of patients with severe asthma has been proven effective in reducing the number of exacerbations, as well as overall health care utilization 2,11]. The process may be conceptualized as three overall steps (Figure 2): 1) confirming the diagnosis, assessing the level of asthma control, and describing the phenotype, 2) assessing potential treatment barriers, for example inhalation technique and adherence, and 3) assessing potential exposures, such as allergens or occupational exposure, and co-morbidities that may contribute to symptoms.

### Step 1. Asthma diagnosis, asthma control, and phenotype

**a. Diagnosis of asthma**

A diagnosis of asthma should be based on a combination of clinical symptoms and the objective demonstration of variable airflow limitation [1,12].

The presence of at least two of the following increases the likelihood of asthma: wheeze, shortness of breath, chest tightness or cough, which *vary* in intensity and over time, and may be *triggered* by factors, such as viral infections, allergens, and non-specific irritants (strong smells, smoke). Isolated symptoms or atypical symptoms decrease the likelihood of asthma [13].

Variable airflow limitation is defined as either reversibility to beta-2-agonist or steroids, peak flow variation, or airway hyperresponsiveness to triggers such as exercise, methacholine, histamine, or mannitol.

The choice of test will depend on the local availability and preference of the clinician: Bronchial challenge tests (BCTs) with methacholine, histamine, mannitol, exercise, and eucapnic voluntary hyperpnea (EVH) have a higher sensitivity than the reversibility test(s) and PEF variation, and may therefore be the preferred initial test [14]. Importantly, it is often necessary to perform more than one diagnostic test in order to confirm the diagnosis objectively, and it may be more effective to include a standard test panel in the diagnostic work-up of severe asthma, for exampl a combination of reversibility testing, PEF diary, and a BCT [14].

In patients with an FEV_1_ < 70%, which prohibits performing a bronchial challenge test, reversibility testing with either beta_2_-agonists or prednisolone and PEF monitoring are the only possible tests. Importantly, patients with severe asthma may have fixed airflow obstruction, which is not reversible. This does *not* preclude a diagnosis of asthma.

The objective confirmation of an asthma diagnosis is of particular importance in patients with difficult asthma, to avoid overtreatment and side effects. A recent study patients managed for severe asthma across five asthma clinics in Denmark, only approximately 50% had their diagnosis confirmed by an objective test, despite having been managed by an asthma specialist for a minimum of 2 years [15].

It is important to note that it is not always possible to verify the diagnosis objectively, but in patients without variable airflow obstruction, down-titration of the dose of ICS followed by retesting should be considered.

**b. Differential diagnoses**

A number of conditions may mimic asthma (Box 1) [1]. These differential diagnoses should be kept in mind when assessing difficult asthma, and the diagnostic work-up performed accordingly. On the other hand, co-morbidities are important contributors to asthma symptoms. Their prevalence and management are described in the sections below.

**Box 1.** Differential diagnoses in severe asthma*.

**COPD** **Bronchiectasis** **Sarcoidosis** **Bronchiolitis obliterans** **Cystic Fibrosis** **Hypersensitivity pneumonitis** **Hypereosinophilic lung diseases** **Tracheobronchomalacia** **Pulmonary embolism** **Coronary Heart Failure** **Endobronchial tumor/foreign body**

*Differential diagnostic conditions which commonly co-exist with asthma are listed under co-morbidities in Table 2.

**c. Asthma control**

The level of asthma control should be routinely assessed, using definitions proposed by the ERS/ATS guidelines on severe asthma: Regarding the level of asthma control, the ERS/ATS guidelines define *uncontrolled* severe asthma as the presence of *at least one* of the following four criteria:

1. Poor symptom control, i.e. Asthma Control Questionnaire (ACQ) consistently > 1.5 or Asthma Control Test (ACT) < 20 (or not well controlled as defined by The Global Initiative for Asthma (GINA) over 3 months of evaluation).
2. Frequent severe exacerbations, defined as needing two or more bursts of systemic corticosteroids (>3 days each) during the previous year.
3. Serious exacerbations, defined as at least one hospitalization, intensive care unit stay, or mechanical ventilation in the previous year.
4. Airflow limitation, defined as a forced expiratory volume in 1 s (FEV_1_) < 80% predicted (in the presence of reduced FEV_1_/forced vital capacity (FVC) while withholding both short- and long-acting bronchidilators.

**d. The phenotype of asthma**

Phenotyping severe asthma is important for predicting treatment response. Phenotypes may be based on the presence and level of inflammation, which may be termed *inflammatory phenotypes* [16], or based on a combination of clinical characteristics, physiological, and inflammatory markers, which may be termed *clinical phenotypes* [17–22].

*Inflammatory phenotypes* are ideally assessed by induced sputum and divided into four groups: eosinophilic asthma (>3% of the total sputum cells), neutrophilic asthma (>61%), mixed granulocytic asthma (both eosinophilia and neutrophilia), and pauci-granulocytic asthma (both eosinophil and neutrophil counts in normal ranges) [23].

Blood eosinophils and exhaled nitric oxide (FeNO) may be used as markers of eosinophilic airway inflammation [24,25]: There is at present no consensus on the most appropriate cut-off for blood eosinophils, but a higher cut-off improves the predictive value of sputum eosinophils: A cut-off of 0.30 x 10^9^ cells/L yields a positive predictive value (PPV) of 66%, which increases to 89% at a cut-off of 0.45 x 10^9^ cells/L [26]. However, the increase in PPV is accompanied by a decrease in sensitivity from 60% to 49% [26]. Similarly for FeNO, a value > 50 ppb indicates a high likelihood of eosinophilic airway inflammation, whereas a FeNO < 25 ppb indicates a low likelihood of eosinophilia [25]. Many patients have intermediate FeNO values (25–50ppb), in these cases the FeNO is less informative. Of note, neutrophilic airway inflammation can only be assessed by induced sputum, as the level of blood neutrophils does not accurately predict airway neutrophils [24].

At present, there is no clear consensus on the definition of clinical phenotypes of asthma [1]. However, the ERS/ATS guidelines state that the following phenotypes of severe asthma are generally recognized: ‘an early-onset allergic phenotype, a later onset obese (primarily female) phenotype and a later onset eosinophilic phenotype’ [1]. A systematic assessment should include information on the following phenotypic characteristics: a) age at onset (early/late); b) IgE-mediated allergy; c) eosinophilia/elevated FeNO; d) fixed airflow obstruction; and e) obesity.

From a pragmatic, clinical perspective, phenotypic traits can be used in the daily clinical care to target-specific treatments: eosinophilia combined with exacerbations predicts effect of anti-IL5 [27] and patients with fixed airflow obstruction and exacerbations may benefit from tiotropium [28]. Furthermore, perennial allergic sensitization, airflow obstruction (FEV1 < 80%) and exacerbations predict effect of anti-IgE [5].

### Step 2. Treatment barriers (adherence, inhalation technique, understanding of asthma)

Treatment barriers are factors that impede the adequate delivery of asthma medications to the airways: They are frequent causes of poor asthma control, and should be addressed routinely in patients with severe asthma. Unfortunately, adherence and inhalation technique are not routinely checked, and there appears to be significant room for improvement: In a recent real life study of patients managed for severe asthma, adherence was only recorded in 30% of patients, and inhalation technique in 19% [15].

**a. Adherence with controller medication**

Poor adherence in asthma is well-documented [29,30], even in difficult asthma: A study of adults referred to a clinic for difficult asthma revealed that 35% of the patients filled their ICS prescriptions less than 50% of the time [31]. Poor adherence with controller medication is associated with poor disease control, and it is estimated that 24% of exacerbations and 60% of asthma-related hospital admissions may be attributed to poor adherence [29,32].

The high proportion of non-adherent patients poses a major challenge for asthma specialists in identifying patients suffering from severe asthma, that is asthma not responding to high-dose medication. This may lead to non-adherent patients inadvertently being prescribed expensive biological medications [19].

Adherence with asthma controller medications should therefore always be checked, preferably by objective assessment, for example filling of prescriptions in electronic registers.

**b. Inhalation technique**

Assessment of the individual patient’s inhaler technique is important [33], and should be checked at each visit [23].Errors in the use of inhalers are common [34,35] and have previously been reported in up to 80% of patients [33]. A further challenge in severe asthma is that patients may be treated with two or more different inhaler types, which may increase the risk of errors. Also, the increasing number of inhalation devices contributes to difficulties for health care providers to have sufficient knowledge in the correct method of use.

A comprehensive, inhaler specific checklist can be downloaded at https://www.nationalasthma.org.au/living-with-asthma/resources/health-professionals/charts/inhaler-technique-checklists.

**c. Patient education**

A shared-care approach to asthma management improves outcomes [36,37], and requires development of a partnership between the asthmatic and the health care providers. Perhaps more than in other diseases patients require good partnerships for adequate treatment, they must learn to recognize potential triggers and symptoms of exacerbations, when to adjust medications and contact health care personnel [13,38].

### Step 3: Identifying potential triggers: exposures and co-morbidities

**a. Exposures**

Identifying potential triggers causing potential asthma symptoms and exacerbations is essential, and this section describes exposures that should be identified as part of the systematic assessment, including allergens, smoking, occupational exposures and medications that may aggravate symptoms.

#### Indoor allergens

Common indoor allergens that may trigger asthma include pets—cats and dogs—house dust mite, moulds, cockroaches, and rodents [39–41], and an important part of the patients history includes asking about possible exposures to indoor allergens such as pets, carpeting, and damp housing.

#### Outdoor allergens

Outdoor allergens such as pollen and mould spores can trigger asthma exacerbations and increase asthma severity in sensitized individuals. Exposure to fungi, especially *Alternaria* and *Cladosporium*, have been associated with an increase in asthma exacerbations and asthma severity [42,43]. Pollen such as grass and birch allergens induce primarily nasal and conjunctival symptoms, but may also exacerbate asthma symptoms [44].

#### Tobacco smoke

Unfortunately, a large proportion of asthma patients have a significant smoking history or are exposed to passive smoking. Several lines of evidence demonstrate that active and passive smoking leads to greater severity of asthma and act as a triggers to exacerbate asthma [45,46]. It appears that the diminished antioxidant capacity along with the oxidant excess associated with tobacco smoke leads to bronchial hyperresponsiveness and worsening airways obstruction [47,48] Due to the accelerated loss of lung function, there is an increased risk of development of COPD in asthmatics who smoke (see ‘Co-morbidities’) [49]. However, it is also important to recognize that smoking per se does not preclude a diagnosis of asthma, and fixed airflow obstruction in severe asthma is not synonymous with COPD. Importantly, asthma patients who develop COPD appear to have a better prognosis than COPD patients without pre-existing asthma [50,51].

#### Occupational exposure

Exposure to multiple occupational allergens have been associated with work-related asthma. These include flour and grain dust (bakers, farmers), isocyanates (painters, automotive industry workers, adhesive workers), formaldehyde (health care workers, hairdressers, cosmetic workers) wood dusts (carpenters) platinum salts (dentists, chemists, photographers, electricians), latex (health care workers, food handlers), and animal allergens (veterinarians, animal breeders and workers, laboratory workers) [52]. In patients with these occupations, as well as patients who report worsening of their symptoms at work, a referral to a specialist in occupational medicine should be considered.

#### Drugs

*Aspirin and other NSAIDs* may trigger severe airway obstruction in up to 10–15% of adults with asthma [53–55]; typically patients with severe and adult onset eosinophilic asthma and comorbid nasal polyposis, a condition termed Samter´s triad, aspirin-exacerbated respiratory disease (AERD) or Non-steroidal anti-inflammatory drugs-exacerbated respiratory disease (N-ERD) [56]. The intolerance is not an IgE mediated allergy and there is no *in vitro* test for diagnosis.

β-receptor antagonists may trigger asthma and non-selective systemic β-receptor antagonists should be avoided in asthmatics. However, even the β_1_-selective antagonists are not completely risk-free; 20% of patients experience symptoms and airflow obstruction after exposure, even to local β-receptor antagonists such as eye drops [57,58].

**b. Co-morbidities associated with severe asthma**

Co-morbidities are a common feature of severe asthma, which may contribute to poor symptom control [59]. Conversely, co-morbidities may be caused by severe asthma; steroid treatment is associated with an increased risk of iatrogenic co-morbidities including obesity, osteoporosis, diabetes, depression, and gastrointestinal reflux [1,60,61](Table 2).

The prevalence, diagnosis, and management of the most common co-morbidities in severe asthma are summarized in Table 1, and described in further detail below:

#### Chronic rhinosinusitis (CRS)

Clinical studies have demonstrated that 50–90% of subjects with severe asthma have signs of chronic rhinosinusitis [62,63]. Patients with CRS may have co-existing nasal polyps (CRSwNP), which is commonly a feature of severe late-onset, eosinophilic asthma, and may furthermore be associated with Aspirin/NSAID sensitivity [56]. Patients with difficult asthma and CRS report more lung symptoms, in particular more cough and sputum, and have more exacerbations [62].

The presence of CRS is assessed on the basis of clinical symptoms such as nasal discharge, in combination with facial pain/pressure or loss of smell, present for a period of at least 12 weeks [64]. The SNOT-22 questionnaire may be used for assessing symptoms [64], and CT of the sinuses and rhinoscopy may be used to assess the presence of sinus inflammation and polyposis [64].

#### Allergic rhinitis

Allergic rhinitis is common in asthma in general, and seems to play a more important role in early onset severe asthma, whereas in late-onset, severe asthma, allergy generally seems to have less clinical impact [59].

#### COPD

Fixed airflow obstruction is a common feature of severe asthma, and it may be impossible to differentiate severe asthma from COPD, in patients with a significant smoking history [65]. Additionally, a significant proportion of severe asthma starts in adulthood, and adult onset asthma in general is associated with low lung function: As many as 46% of adults with asthma onset after the age of 65 years have an FEV1 < 80% prior to being diagnosed with asthma [66]. Hence, asthma may precede COPD, or vice versa, and the clinical impact of this order of development is as yet unclear.

There is no gold standard for differentiating severe asthma from COPD. The GINA guidelines on asthma- COPD overlap syndrome suggest that the likelihood of having either asthma or COPD is based on a thorough assessment of smoking exposure, symptoms (e.g. variable versus persistent symptoms, family history and response to treatment) [67]. Additionally, assessing components of emphysema, for example with DLCO or HRCT may be helpful [50,68].

#### Dysfunctional breathing

Dysfunctional breathing (DB) may accompany asthma or be an asthma mimicker. Defined as ‘chronic changes in breathing pattern that result in dyspnoea and other symptoms, in the absence or in excess of the magnitude of physiological respiratory or cardiac disease’ [69], DB is observed in up to 52% of subjects with difficult asthma. DB seems to be most common in the obese and non-eosinophilic phenotypes of severe asthma [70,71]. Patients typically report very excessive dyspnea at relatively low levels of physical activity, that is dyspnea that is disproportionate with the objective level of asthma severity. Furthermore, extra-pulmonary complaints such as dizziness, fatigue or tingling of the fingers/around the mouth are common. There is no gold standard for diagnosing DB, but patients may be screened using the Nijmegen questionnaire, where a score > 23 indicates an increased likelihood of DB [72].

#### Vocal cord dysfunction

Vocal cord dysfunction (VCD) is an important mimicker of asthma: The vocal cords are inappropriately adducted leading to a sense of dyspnea and shortness of breath with associated stridor rather than wheezing [73]. VCD can present as a single disease but in up to 50 % of patients concomitant VCD and asthma are seen and may complicate diagnosis and treatment in ‘treatment-resistant’ asthma [74]. The diagnosis of VCD may be challenging, as laryngoscopy may be normal when the subject is not experiencing symptoms. No validated protocols for diagnosing VCD exist, however provocation with stimuli such as perfume may be attempted. A typical symptom of VCD is hoarseness during attacks. Patients may be screened for VCD with the Pittsburgh questionnaire [75], and referral to a speech pathologist should be considered. VCD may also be provoked by exercise. Continuous laryngoscopic exercise testing (CLE) may be performed to visualize the larynx during exercise. CLE testing has demonstrated that a large proportion of patients with symptoms suggestive of VCD may demonstrate supraglottic closure of the aryepiglottic folds [76]. The term Inducible Laryngeal Obstruction (ILO) has been proposed, to cover both glottic and supraglottic obstruction, induced by stimuli such as exercise (EILO) or external stimulants [76]. The relative prevalence of glottic (VCD) versus supraglottic obstruction in severe asthma is unknown. CLE may be a useful test for ILO in severe asthma, however protocols for validity and safety for this type of testing have yet to be established.

#### Anxiety and depression

Anxiety and depression are prevalent conditions among patients with severe asthma, and may contribute to symptoms, but also be the result of having a severe and chronic disease [77]. Patients may be screened with questionnaires such as the Hospital depression and Anxiety Scale (HADS) [78,79], although there are as of yet no validated questionnaires for asthma specifically. If anxiety or depression are suspected, the patient should be referred for psychiatric assessment at a center with experience in psychiatric disorders in chronic disease.

#### Obstructive sleep apnea syndrome

Obstructive Sleep Apnea Syndrome (OSAS) is common in severe asthma, and is associated with poor symptom control and frequent exacerbations [80]. A potential vicious cycle may result as OSAS may induce asthma symptoms, and asthma increase the risk of OSAS [70]. Screening of patients for OSAS can be performed with validated questionnaires such as the, STOP-BANG or the Berlin Questionaire [81,82]. If OSAS is suspected, patients should be referred for polysomnography [83].

#### Obesity

Overall, obesity is associated with worse asthma outcomes, especially an increased risk of exacerbations and asthma-related hospitalizations [68,84]. This data may be confounded by other obesity related co-morbidities which also affect asthma exacerbations such as gastroesophageal reflux and sleep apnea [85].

Furthermore, obese asthmatics may have a lower threshold for symptoms: A study comparing asthmatics with higher levels of BMI (>31) compared with lean asthmatics, found that the obese group had a 50% increased risk of asthma exacerbations and a 94% increase in use of rescue inhalers [86,87].

#### Gastroesophageal reflux disease (GERD)

GERD is associated with poor symptom control, as well as more frequent exacerbations in severe asthma [59,88]. Although the causal link is unclear, co-existence of gastroesophageal reflux is very common in patients with asthma [89]. As the diagnosis of GERD may be difficult, with limited sensitivity of both gastric pH monitoring and endoscopy, a trial treatment with PPI may be used as the initial diagnostic step in symptomatic patients [90]. Of note, high use of beta_2_-agonist has been shown to relax the sphincter between the oesophagus and the ventricle, which increases the tendency to reflux, potentially inducing a vicious cycle of high SABA use, reflux and increasing asthma symptoms leading to high SABA use.

#### Bronchiectasis

The prevalence of bronchiectasis in patients with severe asthma is relatively high; with 25–40% of patients having radiological signs of bronchiectasis [91,92], compared to 3% in a population of generally milder asthma [93].

Asthma patients with co-existing bronchiectasis appear to be at higher risk of asthma exacerbations and hospitalizations [93,94]. Bronchiectasis per se may cause obstructive airflow limitation [92], and is thus a also a differential diagnosis of asthma [95]. Furthermore, bronchiectasis increases the susceptibility to infections [95]. Bronchiectasis is associated with allergic bronchopulmonary aspergillosis (ABPA) in severe asthma [96], see below.

#### Allergic bronchopulmonary aspergillosis (ABPA)

ABPA is a hypersensitivity reaction to aspergillus fumigatus [96]: Although a relatively rare condition, ABPA may have significant impact on asthma control. Typically patients experience symptoms of chronic mucus hypersecretion and have an associated accelerated loss of lung function [96].

The most recently diagnostic criteria for ABPA, proposed by ISHAM (The international Society for Human and Animal Mycology), in 2013 [97], are summarized below:

#### Predisposing conditions

- Bronchial asthma, cystic fibrosis

**Obligatory criteria** (both should be present)

- Type I *Aspergillus* skin test positive (immediate cutaneous hypersensitivity to *Aspergillus* antigen) or elevated IgE levels against *Aspergillus fumigatus.*
- Elevated total IgE levels (> 1000 IU/mL) (If the patient meets all other criteria, an IgE value < 1000 IU/mL may be acceptable).

**Other criteria** (at least two of three)

- Presence of precipitating or IgG antibodies against *A. fumigatus* in serum.
- Radiographic pulmonary opacities consistent with ABPA (may be transient).
- Total eosinophil count > 500 cells/μL in steroid naïve patients (may be historical).

Sensitization to aspergillus in severe asthma in the absence of other features of ABPA has been termed severe asthma with fungal sensitization (SAFS) [98]. The management of this condition is somewhat debated: The ERS/ATS guidelines recommend that severe asthma with fungal sensitization, without other characteristics of ABPA, should *not* be treated with anti-fungal agents, due to an overall negative risk-benefit ratio associated with anti-fungal agents [1].

## Management of severe asthma

**a. Non-pharmacological treatments**

### Asthma education

Educating patients in self-management improves health outcomes in severe asthma [8]: Key components of teaching self-management are ensuring adequate knowledge about asthma, providing written action plans, teaching correct inhalation techniques, and encouraging adherence with medications [8]. As doctors often have limited time for the individual patient, specialized asthma nurses may have an important role in providing self-management education.

### Written action plans

Individualized asthma action plans have been demonstrated to improve quality of life [99] and reduce hospital admissions in patients with severe disease. The action plan should include both guidance on maintenance therapy, as well as advice on how to recognize and manage exacerbations [37].

#### Inhaler technique

Many patients with asthma (up to 70–80%) are unable to use their inhaler(s) correctly [13]. Poor inhaler technique leads to reduced asthma control, increased risk of exacerbations and increased adverse effects [33]. Key components ensuring correct inhalation technique are: choosing the most optimal device for the individual patient, avoid different inhaler types when possible and physically demonstrating the use of the inhaler with a placebo inhaler. Furthermore, check inhalation technique with a device-specific checklist on a regular basis, as errors often recur [13].

#### Improving adherence

The first step in improving adherence is to identify the cause of poor adherence, before deciding on an intervention: *Intentional non-adherence* because of fear of side effects or perception that a treatment is unnecessary, or *non-intentional non-adherence*, due to forgetfulness or misunderstanding of instructions, or because of the cost of medications. Finally, some patients have difficulty in being adherent due to difficulties using the device, or because of a regimen with multiple inhalers is overwhelming [13]. Patients with intentional non-adherence due to side effects may need an empathic discussion about the pros and cons of their treatment [36], whereas patients with non-intentional adherence due to forgetfulness may need a more feasible regimen such as once-daily dosing [100], and inhaler reminders [101,102].

#### Removing allergens

In general, allergen avoidance is not recommended as a treatment for asthma. Studies on asthma patients that are sensitized to house dust mite and/or pets, show limited evidence of clinical benefit for asthma with multi-component avoidance and there are no validated methods for identifying those who are likely to benefit [103]. However, in subjects with symptomatic allergy, allergen specific avoidance should be considered if feasible (e.g. removal of furred pets in those with specific sensitization).

In addition, some studies have shown that remediating dampness or mould damages in houses and offices result in reduction of asthma related symptoms and medication use in adults [104].

Another potential method for reducing allergen exposure in for severe allergic asthma, which needs further study, is Temperature-controlled Laminar Airflow (TLA). This treatment is given with a device (Airsonette) that is placed at the patient’s bed and creates an allergy free zone around the patient face in bed, by filtering and lowering the temperature. This treatment has been shown to reduce airway inflammation, and improve quality of life in patients with uncontrolled allergic asthma [105], but has yet to be studied in severe asthma.

#### Smoking cessation

Smoking cessation improves bronchial hyperresponsiveness, as well as symptom control [106], and should be encouraged in all asthma patients.

**b. Pharmacological treatments**

**Figure 3.**
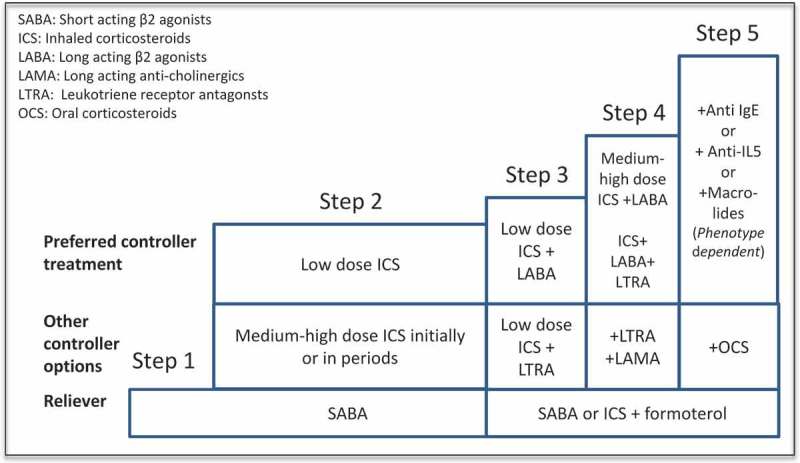
Treatment steps according to GINA guidelines on asthma (REF).

This part of the guidelines are mostly based on the GINA guidelines [107] with some modifications (Figure 3)

#### Step 4 treatment

The pharmacological treatment step 4 is based on medium to high doses of inhaled corticosteroids (ICS) (Figure 3). Patients with severe asthma are per definition treated with high dose ICS (Table 1), combined with a second controller, with inhaled long acting beta-2-agonists (LABA) being the first choice. Other second controllers include leukotriene receptor antagonists (LTRA) [108], tiotropium or theophylline, possibly in combination. The effect of adding in tiotropium is possibly better in ex-smokers than never-smokers [109]. For reliever treatment, short-acting beta-agonists or ICS in combination with formoterol are the two alternatives. In patients with repeated exacerbations using ICS+formoterol is preferred [110]

#### Step 5 treatment

When adequate asthma control with the above treatment is not achieved, oral corticosteroids (OCS) may be added [107], although the evidence for efficacy of long-term OCS in severe asthma is weak. Importantly, the OCS should be used in as low a dose and for the briefest period possible because of the risk of systemic side effects such as osteoporosis [111].

### Allergic asthma

Anti-IgE treatment with omalizumab, a well-studied humanized recombinant IgG_1_ monoclonal antibody with multiple mechanisms of action beyond binding free IgE [112], is an option in severe asthmatics with confirmed perennial allergies (pets or mites). In patients with severe allergic asthma, the add-on treatment with omalizumab has been shown to reduce the risk of exacerbations [113–117], the asthma symptoms [113,114,116,118], and the use of OCS [118] and ICS [115], as well as to improve the quality of life and lung function [113,114,116], without significant safety concerns. These beneficial effects of omalizumab first shown in randomized clinical trials have been confirmed in longer term real-life studies [119–121], and appear to be independent on patients’ comorbidities [118].

Immunotherapy (IT) is presently not recommended in uncontrolled asthma. While in patients with less severe asthma (FEV1 > 70% pred.), HDM-SLIT effectively decreased the number of exacerbations [Virchow JC, et al JAMA 2016], resulting in the inclusion of HDM SLIT in GINA 2017, so far AIT has not been evaluated in severe asthma and hence is an unmet need. The safety of immunotherapy may be improved with addition of Omalizumab [122]. The combined use of Omalizumab followed by IT represents an interesting potential management strategy in severe allergic asthma, and further studies are urgently needed.

### Eosinophilic asthma

Treatment with anti-IL5 has been shown to decrease the risk of exacerbations in patients with eosinophilic asthma [123]. Two compounds are available: mepolizumab which is given subcutaneously and reslizumab which is given intravenously. Both drugs are administered every 4 weeks. In addition to reducing asthma exacerbations, reslizumab has been shown to increase lung function and improve symptom control [124]. Studies indicate an increased effect of anti-IL5 the higher the eosinophilia. [123]. Anti-IL-5 is not indicated unless eosinophil levels are 0.3 x 10^9^/L or greater. The cost of anti-IL-5 is similar to that of omalizumab.

Recent results from the AMAZES study suggest, that low-dose long-term macrolide treatment may improve asthma control in eosinophilic asthma, as well as non-eosinophilic asthma (see below) [125]

### Non-eosinophilic asthma

Macrolides are normally used as antibiotics, but these compounds also have airway biofilm-modifying and anti-inflammatory effects. Treatment with a low dose azithromycin: 250 mg 3 days per week have been shown to reduce the risk of exacerbations in patients with non-eosinophilic asthma (B-Eos≤ 0.2 x 10^9^/L) [126]. In the recently published AMAZES study, low-dose Azithromycin (500 mg 3 days/week for 48 weeks), in addition to usual treatment, reduced exacerbations in uncontrolled eosinophilic as well as non-eosinophilic asthma, as well as asthma-related quality of life [125]. Macrolides reduce neutrophilic airway inflammation [127], but further mechanistic studies are required to understand the mechanisms of action in eosinophilic asthma.

Roflumilast is a phosphodiesterase-4 inhibitor. It is used in severe COPD especially if the patient also has chronic bronchitis. There are some data suggesting a beneficial effect of roflumilast in severe asthma [128].

### Bronchial thermoplasty (BT)

In BT, localized radiofrequency pulses are applied to the airways with bronchoscopy. The treatment has been shown to reduce the risk of asthma exacerbations [129], but is associated with a risk of severe exacerbations in relation to the three bronchoscopies required. Moderate and severe bronchiectasis, very high sputum production, and fixed airflow obstruction with FEV1 levels below 50% predicted are important contraindications for BT. The mechanisms underlying the potential effects of BT are unclear, and may involve effects on the neural innervation of the airways, or on the immune response, as well as the direct effect on airway smooth muscles [130]. Importantly, here is an urgent need for studies that clarify the effect of BT in specific phenotypes of severe asthma, in order to inform clinicians on how to identify patients that will benefit from BT.

**c. Management of co-morbidities**

Management of co-morbidities is important, as they may contribute to the severity of asthma [131]. In general, there is a relative paucity of data on the effect of co-morbidities in asthma .

### Chronic rhinosinusitis (CRS), nasal polyposis

Nasal steroids, systemic steroid treatment, antibiotics, functional endoscopic sinus surgery and aggressive nasal lavage are recommended for the treatment of CRS [64] and improve nasal symptoms. Whereas both medical and surgical interventions have been shown to improve nasal outcomes, there are not convincing data to suggest an improvement in asthma-related outcomes.

In patients with CRS and concomitant nasal polyps (CRSwNP), functional endoscopic surgery (FESS) may reduce asthma symptoms [132], however polyps tend to relapse [133]. Nasal polyps in severe asthma are predominantly eosinophilic, and treatment with anti-IL5 may be a future treatment option [134].

### Allergic rhinitis

Patients with allergic rhinitis and severe asthma are treated with antihistamines, nasal steroids, saline irrigation and specific allergen immunotherapy [135]. Furthermore, anti-leukotrienes may target both upper and lower airways [135,136].

In patients with severe asthma and perennial allergies, anti-IgE (omalizumab) may reduce exacerbation rates [118,137]: Patients with symptoms of allergic rhinitis are more likely to benefit from omalizumab with a substantial reduction in symptoms of rhinitis, as well as those of asthma [138].

### COPD

Little evidence exists on the outcomes of specific treatments in patients with co-existing severe asthma and COPD. However, a multidimensional approach, where treatable traits are identified and targeted is being suggested [139]. This includes addition of LAMA in patients with fixed airflow obstruction, PDE4 inhibitors in patients with significant sputum production and exacerbations, smoking cessation programs, and pulmonary rehabilitation in patients with exertional dyspnea [119].

### Dysfunctional breathing

Breathing retraining may improve symptom control in asthma in general [140], although the effect in severe asthma has not yet been examined. Different protocols for breathing retraining exist, but the general principle is to teach the patient to breathe slowly, through the nose and diaphragmatic breathing instead of using auxiliary respiratory muscles and the apical part of their chest [140,141]. Breathing retraining is performed by physiotherapists and may be combined with relaxation techniques. As these techniques are not performed by all physiotherapists, specific training of local staff may be required.

### Vocal cord dysfunction

VCD may be treated with speech therapy, intended to instruct patients how to relax their vocal cords [142]. The effect of speech therapy has not been studied specifically in severe asthma, but may be attempted in individual patients.

### Anxiety and depression

Psycho-educational interventions such as cognitive behavioural therapy and relaxation techniques may improve asthma control and reduce exacerbations [143], although the evidence is based on small and heterogeneous studies. Access to a psychologist with experience in managing patients with chronic disorders may be helpful in the management of severe asthma [8].

### Obesity

Weight reduction has been shown to improve both symptom control, lung function, and airway hyperresponsiveness in obese asthma patients [144–146]. Even a moderate weight loss of 5–10% is sufficient to achieve clinical improvements [147]. Therefore, obese asthma patients are recommended to lose weight, possibly with the aid of a dietician. Bariatric surgery may be considered in some cases.

### Obstructive sleep apnea (OSAS)

Although the effect has not been studied in difficult asthma per se, CPAP treatment may improve symptom control, lung function, as well as decrease exacerbation rates [148] and airway hyperresponsiveness in asthma in general [149].

### Gastro-esophageal reflux (GERD)

The effect of treating GERD in severe asthma is uncertain: Whereas some studies have demonstrated an effect on symptoms, quality of life and exacerbations, no convincing effect has been demonstrated on lung function and airway hyperresponsiveness [90,150]. Hence, PPI treatment may be considered in the individual patient.

### Bronchiectasis

Management of bronchiectasis aims at improving airway clearance. This can be achieved by reducing airways inflammation, inhalation of hyperosmolar agents and by pulmonary physiotherapy and exercise [151].

Low-dose macrolide treatment may reduce exacerbations in patients with bronchiectasis [151,152]. However, the effect of treating bronchiectasis in severe asthma has not been investigated, and there is a need for further studies.

### Allergic bronchopulmonary aspergillosis

Management of ABPA aims to reduce airway inflammation. This includes treatment with systemic corticosteroids, typically given over months, with an initial dose of 0.5mg/kg of prednisone, tapered over 6–8 weeks, but sometimes over longer periods [96]. Anti-fungal agents may be given in combination with corticosteroids. Itraconazole (200 mg twice daily) is the recommended choice, but voriconazole and posaconazole have also shown a clinical effect in ABPA [96]. Anti-IgE treatment has been shown to be effective in ABPA patients [153] and may be considered in individual patients.

#### Organization of a severe asthma clinic

The assessment of difficult asthma should be done by using a systematic approach [1,4,8] in a specialist center, as this leads to better treatment outcomes and saves costs [2,154,155]. Systematic assessment is also important in ensuring that novel expensive treatments are only used in patients likely to respond. Unfortunately only a minority of subjects considered having severe asthma have been systematically assessed [9]. There are no studies formally assessing the best way to organize the management of severe asthma, but based on current understanding and recommendations by other societies [103] we recommend the following.

### Recommendations for a severe asthma center

A severe asthma center is a specialized unit devoted to diagnosing and the management of severe asthma. Depending on the structure of the health care system it can be organized as a single administrative unit or as collaborative network between professionals from different units. The key elements of a severe asthma center are:

- Severe asthma is the main focus of the center.
- Systematic assessment is used in the diagnostic work-up of severe asthma.
- Several disciplines are represented and a multidisciplinary team is involved in patient management.
- Good facilities for diagnostics and differential diagnostics are available as well as a broad spectrum of treatment modalities.
- The personnel is devoted in continuously improving management of severe asthma and the center is in collaboration with other centers nationally and internationally.
- Clinical research in severe asthma is part of the centers everyday practice and register data are collected to help facilitate research, administration, and assessment of expensive treatments.
- The center(s) coordinate the management of severe asthma and are responsible for education of other health care personnel in the area.

We recommend that a severe asthma center should have the following professionals working within the center or in close collaboration:

- Pulmonologist/allergist and clinical immunologists (preferably at least two persons) specialized in severe asthma and dedicated to lead and develop the center.
- ENT specialists with an interest in rhinology and laryngeal diseases.
  - preferably also a speech pathologist/laryngologist
- Specialist asthma nurse.
- Physiotherapist specialized in dysfunctional breathing and abnormal breathing patterns, capable of assessing and educating patients.
- Radiologist, preferably specialized in thoracic radiology.
- Dietician.
- Psychologist.

Regular Multidisciplinary team (MDT) conferences are useful to ensure an effective decision process in the management of the individual patient. Entering the information gathered during the systematic assessment into a standardized summary sheet may be helpful to ensure an overview during and after the MDT, and might be used in addition as a discharge summary for patients attending the clinic and are referred back locally. Examples are shown in the *online supplementum.*

We recommend that a severe asthma center should have the following equipment/methods available in addition to normal hospital facilities:

- Lung function measurements (spirometry, diffusing capacity, body plethysmography, methods to test for bronchial hyperreactivity (e.g. methacholine, histamine, mannitol), exercise challenges or spiroergometry, impulse oscillometry or forced oscillometry.
- Allergy testing with skin prick testing or serum level of allergen specific IgE and allergen challenge testing.
- Blood tests available, including blood eosinophil counts.
- Tests for airway inflammation: FeNO and preferentially also induced sputum.
- Tests for immunological diseases and/or a clinical immunologist available.
- Bronchoscopy.
- Rhinoscopy.
- Laryngoscopy, which can be performed at rest and during exercise.
- High resolution computed tomography of chest and sinus CT.
- Polysomnography/respiratory polygraphy.

### Organizing severe asthma treatment at national level

In areas where population density is high and long distances are not a major problem, the evaluation and care of severe asthma is usually best organized by establishing several ***severe asthmacenters*** that take care of their own area. In areas with low population density and large geographical distances, where establishing a true severe asthma center may not be possible, we propose that ***severe asthma teams*** may be helpful in improving the treatment of severe asthma. A severe asthma team could be based in local respiratory clinics or other health care units. Such a team would require 1–2 respiratory specialists devoted to severe asthma as well as 1–3 nurses also specialized in severe asthma. It is important that severe asthma teams are devoted to the systematic assessment of severe asthma, interdisciplinary collaboration, and other key elements paralleling a true severe asthma center as much as possible. A severe asthma team should be in close collaboration with the severe asthma center responsible for the area and subjects treated by the team would be included in the register data of the local severe asthma center. Initiation of expensive treatments may be decided by referral to the local severe asthma center or by consultations.

### Referral requirements

**Figure 4.**
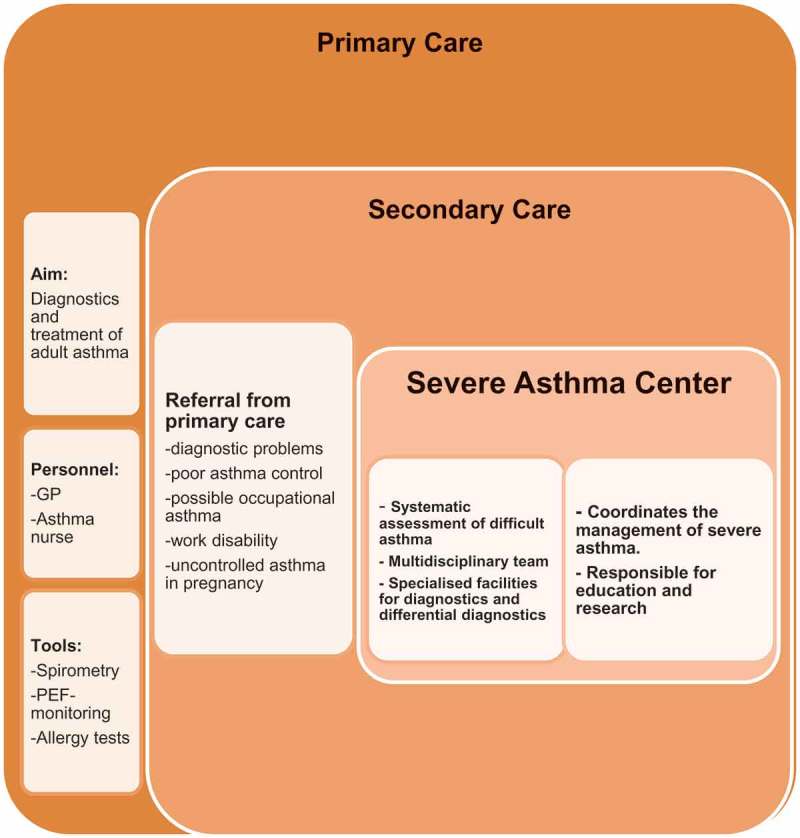
A model for organization of severe asthma management.

The organization of asthma care differs among Nordic countries. In most countries the majority of adult asthmatics are diagnosed and treated in primary health care. However, a patient with a suspicion of or proven severe asthma should be referred to the local severe asthma center or team. As the number of patients with adult and/or adult-onset asthma is high and the burden from these patients is high [156], we propose a two-step model, where the generalist may initially refer the patient to a respiratory specialist, if there is a strong suspicion of or a proven severe asthma, the patient is subsequently referred to the severe asthma center/team (Figure 4). However, depending on the local situation, direct referral from the GP office to the severe asthma center or team may be preferable . A summary of the information that a referral letter to a severe asthma center should include is shown in Box 2.

**Box 2.** The referral letter to the severe asthma center should include as much as available of the following information to facilitate the systematic assessment in specialized severe asthma center/team.

1. **Difficult asthma?** (High dose ICS + second controller): (Y/N) 2. **Asthma control**: Key symptoms:______________________________________________ Exacerbations last 12 months (n):____ 3. **Asthma diagnosis confirmed?** (Y/N). If yes: Which test and when?:_______________________________________________________________ 4. **Current medications**:___________________________________________________ 5. **Results of any treatment trials** (steroid etc.):_________________________________ 6. **Inhalation technique checked and correct**?: _______________________________ 7. **Adherence assessed and acceptable**?: ____________________________________ 8. **Exposures** (Work history, possible exposure to allergens (home, hobbies and work) and other exposures): ___________________________________________________________ 9. **Information on concomitant diseases**: _____________________________________ 10. **Include**: Most recent evaluations of respiratory health and asthma (including chest X-ray, spirometry, peak flow follow-ups and challenge tests as well as laboratory values such blood eosinophils, neutrophils and total immunoglobulin E levels).

*if proper diagnostic evaluations have not been done, relevant diagnostic work-up should be performed by the treating generalist or respiratory specialist before referring the patient to specialized severe asthma clinic (von Bulow, et al. 2017).

### Future directions

The principle target in severe asthma is to achieve optimal control while avoiding side-effects. Reaching this goal is a challenge. However recent advances enabling a better understanding of the mechanisms that drive this disease is changing our approach to how we approach the severe asthmatic, both with regards to diagnosis and treatment. In this context advanced phenotyping and endotyping are crucial [157,158]. This includes collecting clinical information on the individual patient as well as laboratory data such as biomarkers to identify endotypes that underlie phenotypes and make tailored therapy possible [159,160]. Data from both randomized controlled trials [161] (with wide inclusion criteria, followed by subgroup analyses to identify the responder subpopulations [162]), as well as real world evidence should be incorporated [163]. Importantly, the major advances in the management of severe asthma are associated with phenotypes of Type 2-high asthma (e.g. atopic and eosinophilic phenotypes) [163,164], whereas both predictive biomarkers and treatments for asthmatics, whose disease does not show presence of the type 2 inflammation are very limited. Furthermore, it is important to mention that the phenotyping, endotyping, and selecting biomarkers for the personalized approach do not necessarily include single biomolecules, but rather be composed of properly selected constellations or signatures of proteins and other peptides, transcriptomes, genes, microRNAs [164] and metabolites [165,166]. Finally, there is a need for more evidence on the consistency of severe asthma phenotypes, throughout the clinical course of severe asthma [23,159,163,167–169].

Other important issues to be solved regarding severe asthma include gathering information on the prevalence and burden of severe asthma in different countries and regions, where in many cases the data are scarce or absent [170]. Moreover, little is know about the impact of local and socio-cultural factors on the diagnosis and treatment of severe asthma and how this may impact the possibilities to utilize add-on and novel personalized therapies to improve the outcomes in each area [170]. Barriers associated with cost of medication and approval/reimbursement of expensive therapies are an additional challenge and add to this complexity. [170]. Further cost-effectiveness analyses are necessary.

The biological (e.g. anti-IL-5 and anti-IgE antibodies) and other specialized (e.g. bronchial thermoplasty) treatments are expensive. The costs are another factor limiting the number of patients being offered these therapies. National differences in these expensive treatments may differ due to different funding strategies between countries. This undermines the need for international guidelines facilitating the selection of subjects most likely to respond the these novel treatments . On a national level, local severe asthma centers in collaboration with the authorities should provide a list of appropriate criteria for selection of patients to receive novel biological treatments in the respective countries. The decision to initiate these treatments should then be made at the severe asthma centers after a multidisciplinary systematic assessment process, and the efficacy of the treatment should be monitored systematically, to ensure that the treatment is effective and was targeted to the appropriate patients. Finally, national registers of patients treated with expensive biologicals will be important in ensuring evidence on the ‘real-life’ use and efficacy of these drugs.

In conclusion, severe asthma is a difficult clinical challenge, which requires highly specialized care. The development of novel biological treatments, which are expensive, and only effective in selected groups of patients, has further necessitated a high level of expertise among specialists. Local centers for the diagnosis and management of severe asthma are important in achieving these goals.
